# ﻿Tapeworms (Platyhelminthes, Cestoda) from marine chondrichthyans of the Southwestern Atlantic Ocean, and the sub-Antarctic and Antarctic islands: a checklist

**DOI:** 10.3897/zookeys.1163.100485

**Published:** 2023-05-19

**Authors:** Sebastián Franzese, Guillermina García Facal, Adriana Menoret

**Affiliations:** 1 Universidad de Buenos Aires, Facultad de Ciencias Exactas y Naturales, Departamento de Biodiversidad y Biología Experimental, Laboratorio de Sistemática y Biología de Parásitos de Organismos Acuáticos, Ciudad Universitaria, C1428EGA, Buenos Aires, Argentina Universidad de Buenos Aires Buenos Aires Argentina; 2 CONICET – Universidad de Buenos Aires, Instituto de Biodiversidad y Biología Experimental y Aplicada (IBBEA), Buenos Aires, Argentina Universidad de Buenos Aires Buenos Aires Argentina

**Keywords:** Batoids, biodiversity, parasites, sharks, tapeworms, taxonomy

## Abstract

A parasite-host list of cestodes parasitizing chondrichthyans in the Southwest Atlantic off Argentina and surrounding waters of Antarctica is compiled based on the available literature. The list is based on published descriptions and redescriptions of species, and newly collected worms during the current study. A total of 57 valid species belonging to 28 genera of the orders Cathetocephalidea, Diphyllidea, Gyrocotylidea, Lecanicephalidea, Onchoproteocephalidea, Phyllobothriidea, Rhinebothriidea, “Tetraphyllidea”, and Trypanorhyncha is listed. Information on hosts, localities, specimens in collections and comments on tapeworms are also included. A host-parasite list including chimaeras (1 order, 1 genus), batoids (4 orders, 10 genera), and sharks (3 orders, 5 genera) is provided. Tapeworm diversity, distribution range, and host associations are discussed. The cestodes orders Phyllobothriidea and Rhinebothriidea exhibit the highest species richness, with 13 and 12 species, respectively. Onchoproteocephalideans and rhinebothriideans have the broadest geographic distribution in the study area. Regarding hosts, arhynchobatid skates are the group most frequently associated with cestodes. However, further collecting efforts are necessary to understand whether this data reflect the real diversity and host association of these parasites or is a result of a bias in sampling.

## ﻿Introduction

According to Froese and Pauly (2022), more than 500 species of fishes have been registered along the Southwestern Atlantic off Argentina and the sub-Antarctic and Antarctic islands (including South Georgia, Elephant, and Joinville islands), including 100 chondrichthyan species (Table 1) (Gabbanelli et al. 2018; Concha et al. 2019; Froese and Pauly 2022). Since these cartilaginous fishes are the definitive hosts of a great diversity of adult cestodes (Caira and Jensen 2014), it is not uncommon to find a large variety of taxa of tapeworms along the Southwestern Atlantic and the Southern seas.

**Table 1.** Chondrichthyans reported from the Southwestern Atlantic Ocean off Argentina, Río de la Plata estuary, and the sub-Antarctic and Antarctic islands (including South Georgia, Elephant, and Joinville islands).

Subclass	Order	Family	Genera	Species	Sampled chondrichthyan species
**Holocephalii**	Chimaeriformes	Callorhinchidae	1	1	1
		Chimaeridae	1	1	0
Subtotals	**1**	**2**	**2**	**2**	**1**
**Batoidea**	Myliobatiformes	Dasyatidae	2	3	0
		Mobulidae	1	1	0
		Myliobatidae	1	3	2
	Rajiformes	Arhynchobatidae	5	31	19
		Rajidae	2	8	3
	Rhinopristiformes	Pristidae	1	1	0
		Rhinobatidae	1	1	0
		Trygonorrhinidae	1	1	1
	Torpediniformes	Narcinidae	2	3	1
		Torpedinidae	1	1	0
Subtotals	**4**	**10**	**17**	**53**	**26**
**Selachii**	Carcharhiniformes	Carcharhinidae	2	7	1
		Galeocerdonidae	1	1	0
		Scyliorhinidae	2	2	0
		Sphyrnidae	1	3	0
		Triakidae	2	4	3
	Echinorhiniformes	Echinorhinidae	1	1	0
	Hexanchiformes	Hexanchidae	3	3	1
	Lamniformes	Alopiidae	1	1	0
		Carchariidae	1	1	0
		Cetorhinidae	1	1	0
		Lamnidae	3	3	0
	Squaliformes	Dalatiidae	2	2	0
		Etmopteridae	2	6	0
		Somniosidae	3	4	0
		Squalidae	1	3	0
	Squatiniformes	Squatinidae	1	3	1
Subtotals	**6**	**16**	**27**	**45**	6
Totals	**11**	**28**	**46**	**100**	**33**

Complete and accurate species lists are essential for many biological disciplines such as ecology, conservation, and biogeography. Particularly, comprehensive fish cestodes datasets are necessary if we consider the usefulness of these parasites as biological tags for stock identification of their elasmobranch hosts in the context of overfishing and habitat degradation have profoundly altered the populations of marine elasmobranch (Dulvy et al. 2014; Irigoitia et al. 2017; Irigoitia et al. 2022). To date, cestodes from the Southwestern Atlantic, sub-Antarctic, and Antarctic regions were listed only in a few articles. These included a list of fishes and their tapeworms from South America (Alves et al. 2017), a compilation of marine invertebrates from the Argentine Sea focusing on taxonomic information at the generic level only (Bigatti and Signorelli 2018), and a few works about cestodes of Antarctic fishes (Rocka and Zdzitowiecki 1998; Rocka 2003, 2017). The analysis of the endoparasites in Antarctic fishes showed significantly higher values of diversity indices compared to the sub-Antarctic ichthyofauna (Muñoz and Cartes 2020); it would be interesting to consider the diversity of cestodes in a wider context, especially including the Southwestern Atlantic and southern latitudes off Antarctica in a single study. However, no complete work about cestodes from chondrichthyans, with detailed distributional ranges and host associations in this particular area of the Southern Hemisphere has been compiled so far.

In order to facilitate further studies, the main goal of this work is to elaborate a complete checklist of cestodes in chondrichthyan hosts based on summarizing references. The study area includes the Southwestern Atlantic Ocean off Argentina, Río de la Plata estuary, and the surrounding waters of South Georgia and the El­ephant and Joinville islands by surrounding waters of South Georgia, El­ephant and Joinville islands. This list includes information on localities, specimens in collections, and comments about the parasites and their hosts reported in previous works. We have also incorporated information about cestodes described in the last years, which included numerous new records and new localities (Menoret et al. 2017; Franzese and Ivanov 2018, 2020a, b, 2021; Menoret and Ivanov 2021; Franzese et al. 2022; this study).

## ﻿Materials and methods

After an exhaustive bibliographical search, an annotated and revised parasite-host checklist was generated for the adult cestodes from marine chondrichthyans reported between 35°S–63°S. The geographical area considered covers the Southwestern Atlantic Ocean off Argentina (from 35°S southward), Río de la Plata estuary, and surrounding waters off South Georgia, Elephant, and Joinville islands. The cestode species are arranged according to taxonomic categories and are presented alphabetically, followed by data on their hosts, including valid species name, order, family, and synonymous species name used in literature (if available) in parentheses. The information for localities includes location, coordinates in degrees and minutes (if available in the literature), province, and country (where applicable) only for the type locality. The type-host and the type locality refer to data included in the original descriptions of cestodes species. Other hosts and other localities only refer to the records within the study area, including those in the original descriptions as well as those mentioned in redescriptions, other papers and newly collected materials sampled during the present study. Specimens in collections include type material from original descriptions, voucher specimens from redescriptions and new voucher specimens prepared during the present study. Information about the new voucher specimens is in bold.

For the preparation of the figures, estimated coordinates were assigned to those records that lacked such information in the original publication.

Based on the information from the parasite-host checklist, the host-parasite data were subdivided into two inventories, one for batoids and chimaeras and another for sharks. The host species are arranged according to taxonomic categories and presented alphabetically, followed by the data on their parasites.

New vouchers of cestodes were obtained from the spiral intestines of chondrichthyans that had been caught by commercial trawlers between 2009 and 2017. The spiral intestines were fixed in 10% formalin and transferred to 70% ethanol for storage in the Laboratorio de Sistemática y Biología de Parásitos de Organismos Acuáticos (SIBIPOA) of Instituto de Biodiversidad y Biología Experimental y Aplicada (IBBEA, CONICET-UBA). Cestodes were hydrated in a graded ethanol series, stained with Harris’ hematoxylin, dehydrated in a graded ethanol series, cleared in methyl salicylate, and mounted in Canada balsam (Menoret and Ivanov 2021; Franzese et al. 2022).

The accession numbers of the available molecular sequences were taken from the GenBank database, considering only those specimens whose identification is not doubtful.

The classification and valid cestodes names follow Caira and Jensen (2017) and Caira et al. (2022). The classification and valid host names follow Menni and Lucifora (2007), Naylor et al. (2012), Weigmann (2016), Gabbanelli et al. (2018), Concha et al. (2019), Stehmann et al. (2021), and Froese and Pauly (2022). Abbreviations of the collection names used are listed in Table 2.

**Table 2.** Museum abbreviations.

**AHC**	Australian Helminthological Collection, South Australian Museum, Adelaide, Australia
**CHIOC**	Coleção Helmintológica do Instituto Oswaldo Cruz, Rio de Janeiro, Brazil
**BMNH/NHMUK**	Natural History Museum, London, United Kingdom
**HWML**	Harold W. Manter Laboratory of Parasitology, University of Nebraska State Museum, Nebraska, United States of America
**IPCAS**	Institute of Parasitology, Academy of Sciences of the Czech Republic, České Budějovice, Czech Republic
**LRP**	Lawrence R. Penner Parasitology Collection, Department of Ecology and Evolutionary Biology, University of Connecticut, Connecticut, United Stated of America
**MACN-Pa**	Museo Argentino de Ciencias Naturales, Colección Parasitológica, Buenos Aires, Argentina
**MLP**	Colección de Invertebrados, Museo de La Plata, La Plata, Argentina
**MNHNC**	Museo Nacional de Historia Natural de Chile, Santiago, Chile
**MNHNF**	Muséum National d’Histoire Naturelle, Paris, France
**MZPW**	Museum and Institute of Zoology, Polish Academy of Science, Warsaw, Poland
**NMW**	Naturhistorisches Museum Wien, Vienna, Austria
**USNM**	National Museum of Natural History of the Smithsonian Institution, Washington, United States of America
**USNPC**	U. S. National Parasite Collection, Maryland, United States of America, currently incorporated in the UNNM

## ﻿Results

### ﻿Parasite-host checklist

**Order Cathetocephalidea Schmidt & Beveridge, 1990**

**Family Cathetocephalidae Dailey & Overstreet, 1973**

#### Genus *Cathetocephalus* Dailey & Overstreet, 1973

***Cathetocephalusaustralis* Schmidt & Beveridge, 1990**

**Type host.***Carcharhinusbrachyurus* (Günther) (Carcharhiniformes: Carcharhinidae).

**Type locality.** Goolwa, South Australia.

**Other locality.** Off Argentina.

**Specimens in collections.**AHC No. V4123 (holotype); AHC Nos. 17535, 17536 (paratypes).

**References.**Schmidt and Beveridge (1990), Suriano and Labriola (2001a).

##### ﻿Order Diphyllidea Carus, 1863

**Family Echinobothriidae Perrier, 1897**

#### Genus *Coronocestus* Caira, Marques, Jensen, Kutcha & Ivanov, 2013

***Coronocestusnotoguidoi* (Ivanov, 1997)**

*Echinobothriumnotoguidoi* Ivanov, 1997. Syn.

**Type host.***Mustelusschmitti* Springer (Carcharhiniformes: Triakidae).

**Type locality.** Mar del Plata (38°00'S, 57°33'W), Buenos Aires Province, Argentina.

**Specimens in collections.**MLP No. 3893C (holotype); MLP Nos. 3894C (paratypes); USNPC No. 87169 (paratypes).

**GenBank Acc. No.**DQ088034.

**References.**Ivanov (1997), Alarcos et al. (2006), Tyler (2006), Caira et al. (2013b).

**Comments.**Tyler (2006) modified the original description of Ivanov (1997) and added new morphological information based on type material.

#### Genus *Echinobothrium* Van Beneden, 1849

***Echinobothriumacanthocolle* Wojciechowska, 1991**

**Type host.***Amblyrajageorgiana* (Norman) (Rajiformes: Rajidae) (*Rajageorgiana*).

**Type locality.** Shelf near South Georgia, South Atlantic Ocean.

**Specimens in collections.** No specimens were deposited in a public collection.

**References.**Wojciechowska (1991a), Rocka (2003).

**Comments.** Holotype and paratype are in Wojciechowska’s personal collection.

#### Genus *Halysioncum* Caira, Marques, Jensen, Kutcha & Ivanov, 2013

***Halysioncummegacanthum* (Ivanov & Campbell, 1998)**

*Echinobothriummegacanthum* Ivanov & Campbell, 1998. Syn.

**Type host.***Myliobatisgoodei* Garman (Myliobatiformes: Myliobatidae).

**Type locality.** San Antonio Oeste, San Matías Gulf (40°44'S, 64°56'W), Río Negro Province, Argentina.

**Specimens in collections.**MLP No. 3958 (holotype); IPCAS No. C-288 (paratypes); USNM No. 1382674 (paratypes).

**References.**Ivanov and Campbell (1998a), Tyler (2006), Caira et al. (2013b).

**Comments.**Tyler (2006) modified the original description of Ivanov and Campbell (1998a) and added new morphological information based on type material.

***Halysioncumpigmentatum* (Ostrowski de Núñez, 1971)**

*Echinobothriumpigmentatum* Ostrowski de Núñez, 1971. Syn.

**Type host.***Zapteryxbrevirostris* (Müller & Henle) (Rhinopristiformes: Trygonorrhinidae).

**Type locality.** Mar del Plata, Buenos Aires Province, Argentina.

**Specimens in collections.** No specimens were deposited in a public collection.

**References.**Ostrowski de Núñez (1971), Tyler (2006), Caira et al. (2013b).

**Comments.** Holotype and paratypes remain in Ostrowski de Núñez’s personal collection. Tyler (2006) modified the original description of Ostrowski de Núñez (1971) and added new morphological information based on material from the author’s personal collection.

##### ﻿Order Gyrocotylidea Poche, 1926

#### Genus *Gyrocotyle* Diesing, 1850

***Gyrocotylemaxima* Mac Donagh, 1927**

**Type host.** Probably *Callorhinchuscallorynchus* (Linnaeus) (Chimaeriformes: Callorhinchidae) (*Mustelusasterias*).

**Type locality.** Probably off Mar del Plata, Buenos Aires Province, Argentina.

**Specimens in collections.** Instituto Bacteriológico, Buenos Aires.

**Reference.**Mac Donagh (1927).

***Gyrocotylerugosa* Diesing, 1850**

**Type host.***Callorhinchuscallorynchus* (Chimaeriformes: Callorhinchidae).

**Type locality.** Portum Natalensem, South Africa.

**Other locality.** Necochea, Buenos Aires Province.

**Specimen in collections.**NMW No. 2502 (neotype).

**GenBank Acc. Nos.**MW587267, MW587258, MW581656.

**References.**Mac Donagh (1927), Barčák et al. (2021).

**Comments.***Gyrocotylerugosa* has a wide distribution including coastal waters of South America, South Africa, and New Zealand.

##### ﻿Order Lecanicephalidea Hyman, 1951

**Family Aberrapecidae Jensen, Caira, Cielocha, Littlewood & Waeschenbach, 2016**

#### Genus *Aberrapex* Jensen, 2001

***Aberrapexarrhynchum* (Brooks, Mayes & Thorson, 1981)**

*Discobothriumarrhynchum* Brooks, Mayes & Thorson, 1981. Syn.

**Type host.***Myliobatisgoodei* (Myliobatiformes: Myliobatidae).

**Type locality.** Río de la Plata estuary near Montevideo, Uruguay.

**Specimens in collections.**USNPC No. 75722 (holotype); USNPC No. 75723 (paratype); HWML No. 21003 (paratypes).

**References.**Brooks et al. (1981), Jensen (2001).

***Aberrapexludmilae* Menoret, Mutti & Ivanov, 2017**

**Type host.***Myliobatisgoodei* (Myliobatiformes: Myliobatidae).

**Type locality.** San Matias Gulf (40°58'S, 64°56'W), Río Negro Province, Argentina.

**Specimens in collections.**MACN-Pa No 616-1 (holotype); MACN-Pa Nos. 616/2–5 (paratypes); IPCAS No. C-755/1–2 (paratypes); LRP No. 9239 (paratypes).

**Reference.**Menoret et al. (2017).

***Aberrapexsanmartini* Menoret, Mutti & Ivanov, 2017**

**Type host.***Myliobatisgoodei* (Myliobatiformes: Myliobatidae).

**Type locality.** Off Carmen de Patagones (40°42'S, 62°00'W), Buenos Aires Province, Argentina.

**Specimens in collections.**MACN-Pa No. 617/1 (holotype); MACN-Pa Nos. 617/2–12 (paratypes); IPCAS Nos. C-756/1–2 (paratypes); LRP Nos. 9242, 9243 (paratypes).

**Reference.**Menoret et al. (2017).

***Aberrapexvitalemuttiorum* Menoret, Mutti & Ivanov, 2017**

**Type host.***Myliobatisridens* Ruocco, Lucifora, Díaz de Astarloa, Mabragaña & Delpiani (Myliobatiformes: Myliobatidae).

**Type locality.** Off Villa Gesell (37°29'S, 56°45'W), Buenos Aires Province, Argentina.

**Other locality.** Punta Negra, Necochea (38°37'S, 58°51'W), Buenos Aires Province.

**Specimens in collections.**MACN-Pa No 618/1 (holotype); MACN-Pa Nos. 618/2–10 (paratypes); IPCAS Nos. C-757/1–2 (paratypes); LRP Nos. 9240, 9241 (paratypes).

**Reference.**Menoret et al. (2017).

##### ﻿Family Paraberrapecidae Jensen, Caira, Cielocha, Littlewood & Waeschenbach, 2016

#### Genus *Paraberrapex* Jensen, 2001

***Paraberrapexatlanticus* Mutti & Ivanov, 2016**

**Type host.***Squatinaguggenheim* Marini (Squatiniformes: Squatinidae).

**Type locality.** Off Puerto Quequén (38°53'S, 58°27'W), Buenos Aires Province, Argentina.

**Other localities.** Near Río de la Plata estuary (36°21'S, 54°32'W), off Villa Gesell (37°17'S, 56°27'W), off Carmen de Patagones (40°58'S, 62°00'W), Buenos Aires Province. San Matías Gulf (41°03'S, 64°06'W), Río Negro Province.

**Specimens in collections.**MACN-Pa No 618/1 (holotype); MACN-Pa Nos. 618/2–10 (paratypes); IPCAS Nos. C-757/1–2 (paratypes); LRP Nos. 9240, 9241 (paratypes).

**Reference.**Mutti and Ivanov (2016).

##### ﻿Order Onchoproteocephalidea Caira, Jensen, Waeschenbach, Olson & Littlewood, 2014

**Family Onchobothriidae Braun, 1900**

#### Genus *Acanthobothrium* Blanchard, 1848

***Acanthobothriumcarolinae* Franzese & Ivanov, 2020**

**Type host.***Bathyrajamagellanica* (Philippi) (Rajiformes: Arhynchobatidae).

**Type locality.** Coastal waters off Puerto San Julián (49°29'S, 66°11'W), Santa Cruz Province, Argentina.

**Other localities.** Coastal waters off Río Grande (54°01'S, 67°06'W), Tierra del Fuego Province. Namuncurá Marine Protected Area/Burdwood Bank (54°32'S, 60°01'W).

**Specimens in collections.**MACN-Pa No. 716 (holotype); MACN-Pa Nos. 717/1–4, 718/1–3, 719/1–2 (paratypes); IPCAS No. C-838 (paratypes); LRP Nos. 10179–10184 (paratypes).

**Reference.**Franzese and Ivanov (2020a).

***Acanthobothriumdomingae* Franzese & Ivanov, 2020**

**Type host.***Dipturusbrevicaudatus* (Marini) (Rajiformes: Rajidae).

**Type locality.** Coastal waters off Santa Teresita (36°35'S, 54°54'W), Buenos Aires Province, Argentina.

**Other localities.** Coastal waters off Río Grande (53°35'S, 66°37'W), Tierra del Fuego Province. **Coastal waters off Mar del Plata (38°00'S, 56°04'W), Buenos Aires Province** (Table 3).

**Table 3.** Cestodes and their respective hosts collected for this study.

Taxon	Host	Capture coordinates	New locality
Onchoproteocephalidea			
Onchobothriidae			
*Acanthobothrium*			
*A.domingae*	*Dipturusbrevicaudatus*	38°00'S, 56°04'W	Mar del Plata, Buenos Aires
*A.marplatensis*	*Atlantorajacastelnaui*	38°46'S, 57°56'W	Puerto Quequén, Buenos Aires
*A.stefaniae*	*Discopygetschudii*	38°46'S, 57°56'W	Puerto Quequén, Buenos Aires
Rhinebothriidea			
Echeneibothriidae			
*Echeneibothrium*			
*E.williamsi*	*Dipturusbrevicaudatus*	38°46'S, 57°56'W	Puerto Quequén, Buenos Aires
*Notomegarhynchus*			
*N.navonae*	*Atlantorajacastelnaui*	38°46'S, 57°56'W	Puerto Quequén, Buenos Aires

**Specimens in collections.**MACN-Pa No. 720 (holotype); MACN-Pa Nos. 721/1–3, 722/1–9, 723(paratypes); IPCAS No. C-839 (paratypes); LRP Nos. 10185–10195 (paratypes); **MACN-Pa No. 770 (voucher)**.

**Reference.**Franzese and Ivanov (2020a).

***Acanthobothriummarplatensis* Ivanov & Campbell, 1998**

**Type host.***Atlantorajacastelnaui* (Miranda Ribeiro) (Rajiformes: Arhynchobatidae) (*Riorajacastelnaui*).

**Type locality.** Mar del Plata (38°00'S, 57°33'W), Buenos Aires Province, Argentina.

**Other locality. Puerto Quequén (38°46'S, 57°56'W), Buenos Aires Province** (Table 3).

**Specimens in collections.**MLP No. 4025 (holotype); MLP No 4026 (paratype); USNM No. 1382675 (paratypes); BMNH No 1998.2.10.1–2 (paratypes); **MACN-Pa No. 771 (voucher)**.

**Reference.**Ivanov and Campbell (1998b).

***Acanthobothriumstefaniae* Franzese & Ivanov, 2018**

**Type host.***Discopygetschudii* Heckel (Torpediniformes: Narcinidae).

**Type locality.** Coastal waters off Mar Chiquita City (37°46'S, 56°56'W), Buenos Aires Province, Argentina.

**Other localities.** Coastal waters off Villa Gesell (37°29'S, 56°45'W), off San Clemente del Tuyú (35°50'S, 56°18'W), **off Puerto Quequén (38°46'S, 57°56'W)** (Table 3), Buenos Aires Province. Coastal waters off Camarones (45°08'S, 65°19'W), Chubut Province.

**Specimens in collections.**MACN-Pa No 624 (holotype); MACN-Pa Nos. 625/1–6, 626/1–3, 627/1, 628/1–2 (paratypes); IPCAS No. C-786 (paratypes); LRP Nos. 9403–9410 (paratypes); **MACN-Pa No. 772 (voucher)**.

**Reference.**Franzese and Ivanov (2018).

***Acanthobothriumzapterycum* Ostrowski de Núñez, 1971**

**Type host.***Zapteryxbrevirostris* (Rhinopristiformes: Trygonorrhinidae).

**Type locality.** Mar del Plata, Buenos Aires Province, Argentina.

**Other localities.** Coastal waters off Villa Gessel (37°29'S, 56°45'W), La Lucila del Mar (36°38'S, 56°15'W), Puerto Quequén (38°46'S, 57°56'W), Buenos Aires Province. Puerto Pirámides (42°05'S, 62°50'W), Chubut Province.

**Specimens in collections.**MACN-Pa No. 214/1(holotype); MACN-Pa No. 214/1–5 (paratypes); MACN-Pa Nos. 629/1, 630/1–3, 631/1–4, 632/1–4 (vouchers); IPCAS No. C-787 (vouchers); LRP Nos. 9411–9417 (vouchers).

**Reference.**Ostrowski de Núñez (1971), Franzese and Ivanov (2018).

***Acanthobothrium* sp.**

**Hosts.***Bathyrajacousseauae* Díaz de Astarloa & Mabragaña, *Bathyrajamagellanica* (Rajiformes: Arhynchobatidae); *Myliobatisgoodei* (Myliobatiformes: Myliobatidae); *Zapteryxbrevirostris* (Rhinopristiformes: Trygonorrhinidae).

**Localities.** Río de La Plata estuary, Uruguay; Mar del Plata, Buenos Aires Province, Argentina; Malvinas Islands, Southwestern Atlantic Ocean.

**Specimens in collections.**HWML Nos. 20999, 21000.

**References.**Ostrowski de Núñez (1971), Brooks et al. (1981), Beer et al. (2019).

**Comments.**Ostrowski de Núñez (1971) registered *Acanthobothrium* sp. from *Z.brevirostris* in Mar del Plata. Brooks et al. (1981) reported two specimens of *Acanthobothrium* sp. from *M.goodei* at Río de la Plata, which could be a different species. They pointed out that one of these specimens could correspond to the same species reported by Ostrowski de Núñez (1971) in *Z.brevirostris*. Beer et al. (2019) reported *Acanthobothrium* sp. from *B.cousseauae* and *B.magellanica* off Malvinas Islands. The deposited material only corresponds to the specimens studied by Brooks et al. (1981).

#### Genus *Onchobothrium* de Blainville, 1828

***Onchobothriumantarcticum* Wojciechowska, 1990**

**Type host.***Bathyrajaeatonii* (Günther) (Rajiformes: Arhynchobatidae).

**Type locality.** shelf around Joinville Island in Bransfield’s Strait, Antarctica.

**Specimens in collections.**MZPW No. 1805 (holotype); MZPW No. 1806 (paratype); BMNH 1989.4.19.1 (paratype).

**References.**Wojciechowska (1990a), Rocka (2003, 2017).

##### ﻿Order Phyllobothriidea Caira, Jensen, Waeschenbach, Olson & Littlewood, 2014

**Family Phyllobothriidae Braun, 1900**

#### Genus *Crossobothrium* Linton, 1889

***Crossobothriumantonioi* Ivanov, 2009**

**Type host.***Notorynchuscepedianus* (Péron) (Hexanchiformes: Hexanchidae).

**Type locality.** Puerto Quequén (38°32'S, 58°42'W), Buenos Aires Province, Argentina.

**Specimens in collections.**MACN-Pa No. 493/1 (holotype); MACN-Pa Nos. 493/2–6 (paratypes).

**Reference.**Ivanov (2009).

***Crossobothriumpequeae* Ivanov, 2009**

**Type host.***Notorynchuscepedianus* (Hexanchiformes: Hexanchidae).

**Type locality.** Puerto Quequén (38°32'S, 58°42'W), Buenos Aires Province, Argentina.

**Specimens in collections.**MACN-Pa No. 494/1 (holotype); MACN-Pa Nos. 494/2–6 (paratypes).

**Reference.**Ivanov (2009).

#### Genus *Guidus* Ivanov, 2006

***Guidusantarcticus* (Wojciechowska, 1991)**

*Marsupiotbothriumantarcticum* Wojciechowska, 1991. Syn.

**Type host.***Bathyrajamaccaini* Springer (Rajiformes: Arhynchobatidae).

**Other host.***Bathyrajaeatonii* (Rajiformes: Arhynchobatidae).

**Type locality.** Shelf around Joinville Island, Antarctica.

**Specimens in collections.**MZPW No. 1817 (holotype); BMNH No. 1992.1.6.31 (paratype).

**References.**Wojciechowska (1991a), Rocka (2003), Ivanov (2006).

***Guidusargentinense* Ivanov, 2006**

**Type host.***Bathyrajabrachyurops* (Fowler) (Rajiformes: Arhynchobatidae).

**Type locality.** Coastal waters off Buenos Aires Province (37°06'S, 54°20'W), Argentina.

**Other localities.** Off Bahía Blanca (39°34'S, 56°16'W), Buenos Aires Province. Namuncurá Marine Protected Area/Burdwood Bank (54°44'S, 59°56'W).

**Specimens in collections.**MACN-Pa No. 432/1 (holotype); MACN-Pa Nos. 432/2–7 (paratypes); USNM No. 1393041 (paratypes); MACN-Pa Nos. 750–751 (vouchers).

**References.**Ivanov (2006), Menoret and Ivanov (2021).

***Guidusfrancoi* Menoret & Ivanov, 2021**

**Type host.***Bathyrajamagellanica* (Rajiformes: Arhynchobatidae).

**Type locality.** Off Río Grande (53°56'S, 66°04'W), Tierra del Fuego Province, Argentina.

**Other localities.** Off Puerto San Julián (49°29'S, 66°11'W), Santa Cruz Province. Off Río Grande (54°30'S, 65°13'W; 54°24'S, 63°57'W; 54°01'S, 67°06'W; 53°55'S, 67°05'W; 53°36'S, 67°39'W), Tierra del Fuego Province.

**Specimens in collections.**MACN-Pa No. 739 (holotype); MACN-Pa Nos. 740/1–3, 741/1, 744, 745, 746/1–2, 740/4, 741/2–3, 742/1–3, 743, 746/3–7 (paratypes); IPCAS No. C-887 (paratypes).

**Reference.**Menoret and Ivanov (2021).

***Guidusmagellanicus* Menoret & Ivanov, 2021**

**Type host.***Bathyrajamagellanica* (Rajiformes: Arhynchobatidae).

**Type locality.** Off Río Grande (54°01'S, 67°06'W), Tierra del Fuego Province, Argentina.

**Other localities.** Off Puerto San Julian (49°29'S, 66°11'W), Santa Cruz Province.

**Specimens in collections.**MACN-Pa No. 747 (holotype); MACN-Pa Nos. 748/1–2, 749/1–2 (paratypes); IPCAS No. C-888 (paratypes).

**Reference.**Menoret and Ivanov (2021).

***Guidus* sp.**

**Host.***Bathyrajamultispinis* (Norman) (Rajiformes: Arhynchobatidae).

**Locality.** Malvinas Islands Shelf, Southwestern Atlantic Ocean.

**Reference.**Beer et al. (2019).

**Comments.** These specimens were studied by Beer et al. (2019) at a molecular rather than morphological level, without reaching an identification at the specific level.

#### Genus *Orygmatobothrium* Diesing, 1863

***Orygmatobothriumjuani* Ivanov, 2008**

**Type host.***Mustelusfasciatus* (Garman) (Carcharhiniformes: Triakidae).

**Type locality.** Puerto Quequén (38°32'S, 58°42'W), Buenos Aires Province, Argentina.

**Specimens in collections.**MACN-Pa No. 445/1 (holotype); MACN-Pa Nos. 445/2–6 (paratypes).

**Reference.**Ivanov (2008).

***Orygmatobothriumschmitti* Suriano & Labriola, 2001**

**Type host.***Mustelusschmitti* (Carcharhiniformes: Triakidae).

**Type locality.** Mar del Plata (38°00'S, 57°33'W), Buenos Aires Province, Argentina.

**Other locality.** Puerto Quequén (38°32'S, 58°42'W), Buenos Aires Province.

**Specimens in collections.**MACN-Pa Nos. 382/1–2 (holotype and paratype); MNHN 20HG:158 CIX, MNHN 20HG:159 CIX (paratypes); MACN-Pa Nos. 444/1–5 (vouchers).

**References.**Ostrowski de Núñez (1973), Suriano and Labriola (2001b), Alarcos et al. (2006), Ivanov (2008).

**Comments.**Ostrowski de Núñez (1973) redescribed *O.velamentum* based on material collected in Mar del Plata. Later, Ivanov (2008) reassigned these specimens to *O.schmitti*.

#### Genus *Phyllobothrium* Van Beneden, 1850

***Phyllobothrium* sp.**

**Hosts.***Sympterygiabonapartii* Müller & Henle (Rajiformes: Rajidae) (as *Psammobatismicrops* in Ostrowski de Núñez [1971]), *Myliobatisgoodei* (Myliobatiformes: Myliobatidae), *Zapteryxbrevirostris* (Rhinopristiformes: Trygonorrhinidae).

**Localities.** Mar del Plata, Argentina. Río de la Plata estuary near Montevideo, Uruguay.

**Specimen in collections.**HWML 21001.

**References.**Ostrowski de Núñez (1971), Brooks et al. (1981).

**Comments.**Ostrowski de Núñez (1971) registered *Phyllobothrium* sp. from *S.bonapartii* and *Z.brevirostris* in Mar del Plata. Brooks et al. (1981) reported ten specimens of *Phyllobothrium* sp. from *M.goodei* at the Río de la Plata estuary. The deposited material only corresponds to the specimens studied by Brooks et al. (1981).

#### Genus *Rockacestus* Caira, Bueno & Jensen, 2021

***Rockacestusarctowskii* (Wojciechowska, 1991)**

*Phyllobothriumarctowskii* Wojciechowska, 1991, *Anthocephalumarctowskii* Rocka & Zdzitowiecki, 1998. Syns.

**Type host.***Bathyrajaarctowskii* (Dollo) (Rajiformes: Arhynchobatidae) (*Bathyraja* sp. 2).

**Type locality.** Admiralty Bay, environs of the South Shetlands, Antarctica.

**Other locality.** Shelf near Elephant Island, Antarctica.

**Specimens in collections.**MZPW No. 1814 (holotype); BMNH No. 1992.1.6.30 (paratypes).

**References.**Wojciechowska (1991b), Rocka (2003, 2017), Caira et al. (2021).

**Comments.**Rocka (2017) established the name *Rajicestus* Rocka & Laskowski, 2017 for cestodes from Antarctic and sub-Antarctic skates described originally in Wojciechowska (1991b) as members of *Phyllobothrium*. Unfortunately, no generic diagnosis or type species was designated; therefore, the name *Rajicestus* is unavailable. Regarding host identification, Stehmann et al. (2021) assigned specimens of *Bathyraja* sp. 2 to *Bathyrajaarctowskii*, a wide-ranging, circum-Antarctic species locally common in the Atlantic sector of the Southern Ocean.

***Rockacestusconchai* Caira, Bueno & Jensen, 2021**

**Type host.***Bathyrajaalbomaculata* (Norman) (Rajiformes: Arhynchobatidae).

**Type locality.** Malvinas Islands (48°39'S, 60°44'W), Southwestern Atlantic Ocean.

**Other locality.** Malvinas Islands (49°38'S, 59°50'W).

**Specimens in collections.** NHMUK No. 2020.12.17.1 (holotype); USNM Nos. 1638654, 1638655 (paratypes); LRP Nos. 10293, 10294 (paratypes); LRP Nos. 10279–10281 (SEM vouchers).

**GenBank Acc. No.**MW419959.

**Reference.**Caira et al. (2021).

***Rockacestusgeorgiensis* (Wojciechowska, 1991)**

*Phyllobothriumgeorgiense* Wojciechowska, 1991, *Anthocephalumgeorgiense* Rocka & Zdzitowiecki, 1998. Syns.

**Type host.***Amblyrajageorgiana* (Rajiformes: Rajidae) (*Rajageorgiana*).

**Type locality.** Shelf around South Georgia, South Atlantic Ocean.

**Specimens in collections.**MZPW No. 1812 (holotype); No. BMNH No. 1992.1.6.27 (paratype).

**References.**Wojciechowska (1991b), Rocka (2003, 2017), Caira et al. (2021).

**Comments.**Rocka (2017) established the name *Rajicestus* for cestodes from Antarctic and sub-Antarctic skates described originally in Wojciechowska (1991b) as members of *Phyllobothrium*. Unfortunately, no generic diagnosis or type species was designated; therefore, the name *Rajicestus* is unavailable.

***Rockacestusrakusai* (Wojciechowska, 1991)**

*Phyllobothriumrakusai* Wojciechowska, 1991, *Anthocephalumrakusai* Rocka & Zdzitowiecki, 1998. Syns.

**Type host.***Bathyrajamaccaini* (Rajiformes: Arhynchobatidae).

**Type locality.** Shelf around Elephant Island and Joinville Island in Bransfield Strait, Antarctica.

**Specimens in collections.**MZPW No. 1816 (holotype); BMNH No. 1992.1.6.28 (paratype).

**References.**Wojciechowska (1991b), Rocka (2003, 2017), Caira et al. (2021).

**Comments.**Rocka (2017) established the name *Rajicestus* for cestodes from Antarctic and sub-Antarctic skates described originally in Wojciechowska (1991b) as members of *Phyllobothrium*. Unfortunately, no generic diagnosis or type species was designated; therefore, the name *Rajicestus* is unavailable.

***Rockacestussiedleckii* (Wojciechowska, 1991)**

*Phyllobothriumsiedleckii* Wojciechowska, 1991, *Anthocephalumsiedleckii* Rocka & Zdzitowiecki, 1998. Syns.

**Type host.***Bathyrajaeatonii* (Rajiformes: Arhynchobatidae).

**Type locality.** Shelf around Elephant Island and Joinville Island in Bransfield Strait, Antarctica.

**Specimens in collections.**MZPW No. 1815 (holotype); BMNH No. 1992.1.6.29 (paratype).

**References.**Wojciechowska (1991b), Rocka (2003, 2017), Caira et al. (2021).

**Comments.**Rocka (2017) established the name *Rajicestus* for cestodes from Antarctic and sub-Antarctic skates described originally in Wojciechowska (1991b) as members of *Phyllobothrium*. Unfortunately, no generic diagnosis or type species was designated; therefore, the name *Rajicestus* is unavailable.

**Phyllobothriidea gen. sp.**

**Hosts.***Amblyrajadoellojuradoi* (Pozzi), *Bathyrajaalbomaculata*, *B.brachyurops*, *B.cousseauae*, *B.macloviana* (Norman), *B.magellanica*, *B.multispinis*, *B.scaphiops* (Norman), *Dipturuschilensis* (Guichenot), *Psammobatis* sp. 3, *Psammobatis* sp. 2.

**Locality.** Malvinas Islands Shelf, Southwestern Atlantic Ocean.

**Reference.**Beer et al. (2019).

**Comments.**Beer et al. (2019) studied these specimens at the molecular rather than the morphological level, without reaching generic or specific identification. Caira et al. (2021) noted that the specimens of Phyllobothriidea gen. sp. found by Beer et al. (2019) could correspond to the genus *Rockacestus*; however, further molecular and morphological studies are necessary to identify them at the specific level. Beer et al. (2019) also pointed out the presence of Phyllobothriidea gen. sp. parasitizing *D.chilensis*. Nevertheless, the distribution of *D.chilesis* is restricted to the Pacific Ocean; therefore, this record is based on a misidentification of the host (Concha et al. 2019).

##### ﻿Order Rhinebothriidea Healy, Caira, Jensen, Webster & Littlewood, 2009

**Family Echeneibothriidae de Beauchamp, 1905**

#### Genus *Echeneibothrium* van Beneden, 1850

***Echeneibothriumcristinae* Franzese, 2022**

**Type host.***Bathyrajacousseauae* (Rajiformes: Arhynchobatidae).

**Type locality.** Isla de los Estados (54°25'S, 65°18'W), Tierra del Fuego Province, Argentina.

**Specimens in collections.**MACN-Pa No. 734 (holotype); MACN-Pa Nos. 735/1–5, 736/1–23 (paratypes).

**Reference.**Franzese et al. (2022).

***Echeneibothriummultiloculatum* Carvajal & Dailey, 1975**

**Type host.***Dipturuschilensis* (Rajiformes: Rajidae) (*Rajachilensis*).

**Other host.***Dipturusbrevicaudatus* (Rajiformes: Rajidae).

**Type locality.** Between Papudo and Talcahuano (between 32°28'S and 37°15'S), Chile.

**Other localities.** Mar de Ajó (36°34'S, 54°39'W), Mar del Plata (38°05'S, 56°58'W), Quequén (38°35'S, 58°39'W), Buenos Aires Province. San Jorge Gulf (46°13'S, 66°26'W), Santa Cruz Province. Tolhuin (54°29'S, 65°59'W), Río Grande (53°31'S, 67°48'W), Tierra del Fuego Province.

**Specimens in collections.**USNM No. 1368523 (holotype); USNM No. 1368524 (paratypes); MACN-Pa Nos. 737/1–10, 738, 739, 740/1–8 (vouchers).

**GenBank Acc. Nos.**MZ594651, MH688748, KY569546, KY569547, KY569548, KY569549.

**References.**Carvajal and Dailey (1975), Franzese et al. (2022).

***Echeneibothriumwilliamsi* Carvajal & Dailey, 1975**

**Type host.***Dipturuschilensis* (Rajiformes: Rajidae) (*Rajachilensis*).

**Other host.***Dipturusbrevicaudatus* (Rajiformes: Rajidae).

**Type locality.** Between Papudo and Talcahuano (between 32°28'S and 37°15'S), Chile.

**Other localities.** San Jorge Gulf (46°13'S, 66°26'W), Santa Cruz Province. Tolhuin (54°29'S, 65°59'W), Río Grande (53°31'S, 67°48'W), Tierra del Fuego Province. **Puerto Quequén (38°46'S, 57°56'W), Buenos Aires Province** (Table 3).

**Specimens in collections.**USNM No. 1368521 (holotype); USNM No. 1368522 (paratypes); MACN-Pa Nos. 741/1–14, 742/1–4, 743, **773** (vouchers).

**GenBank Acc. Nos.**MZ594641, MH688742, KY569542, KY569543, KY569544, KY569545.

**References.**Carvajal and Dailey (1975), Franzese et al. (2022).

***Echeneibothrium* sp.**

**Hosts.***Bathyrajaalbomaculata*, *B.brachyurops*, *B.cousseauae*, *B.griseocauda* (Norman), *B.macloviana*, *B.multispinis*, *B.scaphiops* (Rajiformes: Arhynchobatidae).

**Locality.** Malvinas Islands, Southwestern Atlantic Ocean.

**Reference.**Beer et al. (2019).

**Comments.** These specimens were studied by Beer et al. (2019) at a molecular rather than morphological level and did not manage to reach an identification at a specific level. Franzese et al. (2022) noted that the specimens of *Echeneibothrium* sp. found in *B.cousseauae* by Beer et al. (2019) at Malvinas Islands could correspond to *E.cristinae*. Considering that the remaining species of *Bathyraja* have not been recorded as hosts for *Echeneibothrium* and that most marine rhinebothriideans show a high degree of specificity to their definitive hosts, Franzese et al. (2022) supposed that some *Echeneibothrium* specimens reported by Beer et al. (2019) could be new species. However, further morphological studies are necessary to identify them at a specific level. Beer et al. (2019) also pointed out the presence of *Echeneibothrium* and *Echeneibothrium* sp. 2 parasitizing *D.chilensis* at Malvinas Islands; however, the distribution of *D.chilesis* is restricted to the Pacific Ocean, i. e. this record has been based on a host misidentification.

#### Genus *Notomegarhynchus* Ivanov & Campbell, 2002

***Notomegarhynchusnavonae* Ivanov & Campbell, 2002**

**Type host.***Atlantorajacastelnaui* (Rajiformes: Arhynchobatidae).

**Type locality.** Mar del Plata (38°00'S, 57°33'W), Buenos Aires Province, Argentina.

**Other locality. Puerto Quequén (38°46'S, 57°56'W), Buenos Aires Province** (Table 3).

**Specimens in collections.**MACN-Pa No. 404/1 (holotype); MACN-Pa Nos. 404/2–3 (paratypes); USNM No.1387025 (paratypes); **MACN-Pa No. 774 (voucher)**.

**Reference.**Ivanov and Campbell (2002).

***Notomegarhynchusshetlandicum* (Wojciechowska, 1990)**

*Pseudanthobothriumshetlandicum* Wojciechowska, 1990. Syn.

**Type host.***Bathyrajaeatonii* (Rajiformes: Arhynchobatidae).

**Other host.***Bathyrajamaccaini* (Rajiformes: Arhynchobatidae).

**Specimens in collections.**MZPW No. 1810 (holotype); MZPW No. 1811 (paratypes); BMNH No. 1989.4.19.3 (paratypes).

**Type locality.** South Shetlands region, Joinville shelf, Elephant Island

Shelf, and Admiralty Bay, Antarctica.

**References.**Wojciechowska (1990b), Ivanov and Campbell (2002), Rocka (2003, 2017).

#### Genus *Pseudanthobothrium* Baer, 1956

***Pseudanthobothriumnotogeorgianum* Wojciechowska, 1990**

**Type host.***Amblyrajageorgiana* (Rajiformes: Rajidae) (*Rajageorgiana*).

**Type locality.** South Georgia area, South Atlantic Ocean.

**Specimens in collections.**MZPW No. 1807 (holotype); MZPW Nos. 1808–1809 (paratypes); BMNH No. 1989.4.19.2 (paratypes).

**References.**Wojciechowska (1990b), Rocka (2003, 2017).

***Pseudanthobothriumminutum* Wojciechowska, 1991**

**Type host.***Bathyrajaeatonii* (Rajiformes: Arhynchobatidae).

**Type locality.** Elephant Island, Antarctica.

**Specimens in collections.** No specimens were deposited in a public collection.

**References.**Wojciechowska (1991a), Rocka (2003, 2017).

**Comments.** Type specimens are in Wojciechowska’s personal collection.

***Pseudanthobothrium* sp.**

**Host.***Amblyrajadoellojuradoi* (Rajiformes: Rajidae).

**Locality.** Malvinas Islands, South Atlantic Ocean.

**Reference.**Beer et al. (2019).

**Comments.**Beer et al. (2019) indicated the presence of *Pseudanthobothrium* sp. and *Pseudanthobothrium* sp. 2 parasitizing *A.doellojuradoi* at Malvinas Islands.

##### ﻿Family Rhinebothriidae Euzet, 1953

#### Genus *Rhinebothrium* Linton, 1890

***Rhinebothriumchilensis* Euzet & Carvajal, 1973**

**Type host.***Sympterygialima* (Poeppig) (Rajiformes: Arhynchobatidae) (*Psammobatislima*).

**Other host.***Sympterygiabonapartii* (Rajiformes: Arhynchobatidae).

**Type locality.** North coast of Chile.

**Other localities.** Estuary of Bahía Blanca (38°45'S, 62°15'W), Villa Gesell, Necochea, El Rincón, Buenos Aires Province, Argentina. San Matías Gulf, Río Negro Province, Argentina. San Jorge Gulf, Santa Cruz Province, Argentina. Río de La Plata estuary, Uruguay.

**Specimens in collections.**MNHNC No. 20005 (holotype); MNHNF Nos. Sb 267, Sb 268 (paratypes).

**References.**Euzet and Carvajal (1973), Tanzola et al. (1998), Irigoitia et al. (2017).

##### ﻿Genus *Scalithrium* Ball, Neifar & Euzet, 2003

***Scalithriumivanovae* Franzese, 2021**

**Type host.***Atlantorajaplatana* (Günther) (Rajiformes: Arhynchobatidae).

**Type locality.** San Matías Gulf (41°11'S, 64°03'W), Río Negro Province, Argentina.

**Specimens in collections.**MACN-Pa No. 762 (holotype); MACN-Pa Nos. 763/1–4, 764/1–7, 765/1–3 (paratypes); IPCAS No. C-897 (paratypes).

**Reference.**Franzese and Ivanov (2021).

***Scalithriumkirchneri* Franzese & Ivanov, 2021**

**Type host.***Riorajaagassizii* (Müller & Henle) (Rajiformes: Arhynchobatidae).

**Type locality.** Continental shelf waters off San Clemente del Tuyú (36°12'S, 55°20'W), Buenos Aires Province, Argentina.

**Other locality.** Continental shelf waters off Quequén (39°56'S, 58°20'W), Buenos Aires Province.

**Specimens in collections.**MACN-Pa No. 757 (holotype); MACN-Pa Nos. 758/1–13, 759, 760/1–3, 761(paratypes); IPCAS No. C-896 (paratypes).

**Reference.**Franzese and Ivanov (2021).

### ﻿Genus incertae sedis and other forms with uncertain family allocations

#### Genus *Semiorbiseptum* Franzese & Ivanov, 2020

***Semiorbiseptumalfredoi* Franzese & Ivanov, 2020**

**Type host.***Psammobatisnormani* McEachran (Rajiformes: Arhynchobatidae).

**Type locality.** Coastal waters off Mar de Ajó (36°34'S, 54°39'W), Buenos Aires Province, Argentina.

**Other localities.** Coastal waters off Pinamar (37°12'S, 54°53'W), Buenos Aires Province. Caleta Olivia (46°23'S, 64°20'W), Santa Cruz Province.

**Specimens in collections.**MACN-Pa No. 706 (holotype); MACN-Pa Nos. 707/1–5, 708/1–3, 709, 710, 711/1–2 (paratypes); IPCAS No. C-837/1 (paratypes).

**Reference.**Franzese and Ivanov (2020b).

***Semiorbiseptummariae* Franzese & Ivanov, 2020**

**Type host.***Psammobatisrudis* Günther (Rajiformes: Arhynchobatidae).

**Other host.***Psammobatisnormani* (Rajiformes: Arhynchobatidae).

**Type locality.** Coastal waters off Isla de los Estados (54°30'S, 65°13'W), Tierra del Fuego Province, Argentina.

**Other localities.** Coastal waters off Río Grande (53°34'S, 66°32'W), Tierra del Fuego Province. Coastal waters off Miramar (39°34'S, 56°16'W), Buenos Aires Province.

**Specimens in collections.**MACN-Pa No. 701 (holotype); MACN-Pa Nos. 702/1–4, 703, 704/1–13, 705 (paratypes); IPCAS No. C-836/1 (paratypes).

**Reference.**Franzese and Ivanov (2020b).

**Rhinebothriidea gen. sp.**

**Hosts.***Psammobatis* sp. 1, *Psammobatis* sp. 2, *Psammobatis* sp. 3 (Rajiformes: Arhynchobatidae).

**Locality.** Malvinas Islands, Southwestern Atlantic Ocean.

**References.**Beer et al. (2019).

**Comments.** These cestode specimens were studied by Beer et al. (2019) at a molecular rather than morphological level.

##### ﻿Order “Tetraphyllidea” van Beneden, 1850

**Clade 2**

#### Genus *Anthobothrium* van Beneden, 1850

***Anthobothriumgaleorhini* Suriano, 2002**

**Type host.***Galeorhinusgaleus* (Linnaeus) (Carcharhiniformes: Triakidae).

**Type locality.** Puerto Madryn (42°43'S, 65°00'W), Chubut Province, Argentina.

**Specimens in collections.**MLP No. 4942 (holotype); MNHN No. 37G (paratype).

**Reference.**Suriano (2002).

***Anthobothrium* sp.**

**Host.***Bathyrajaarctowskii* (Rajiformes: Arhynchobatidae) (*Bathyraja* sp. 2).

**Locality.** Drake Strait near King George Island and environs of Elephant Island, Antarctica.

**Reference.**Wojciechowska (1991a, b).

**Comments.** Specimens remain in Wojciechowska’s personal collection. Regarding host identification, Stehmann et al. (2021) assigned specimens of *Bathyraja* sp. 2 to *Bathyrajaarctowskii*, a wide-ranging, circum-Antarctic species locally common in the Atlantic sector of the Southern Ocean.

##### ﻿Family Calliobothriidae Perrier, 1897

#### Genus *Calliobothrium* van Beneden, 1850

***Calliobothriumaustralis* Ostrowski de Núñez, 1973**

**Type host.***Mustelusschmitti* (Carcharhiniformes: Triakidae).

**Type locality.** Mar del Plata, Buenos Aires Province, Argentina.

**Other locality.** Puerto Quequén (38°32'S, 58°42'W), Provincia de Buenos Aires.

**Specimens in collections.** MACN No. 409/1 (holotype); MACN Nos. 405/1–4 (vouchers); USNPC No. 92398 (voucher).

**GenBank Acc. Nos.**KP128030, KP128031.

**References.**Ostrowski de Núñez (1973), Ivanov and Brooks (2002), Alarcos et al. (2006).

**Comments.**Ivanov and Brooks (2002) redescribed *C.australis* based on the material studied originally by Ostrowski de Núñez (1973), who considered this species a subspecies of *C.verticillatum*.

#### Genus *Symcallio* Bernot, Caira & Pickering, 2015

***Symcalliobarbarae* (Ivanov & Brooks, 2002)**

*Calliobothriumbarbarae* Ivanov & Brooks, 2002. Syn.

**Type host.***Mustelusschmitti* (Carcharhiniformes: Triakidae).

**Type locality.** Puerto Quequén (38°32'S, 58°42'W), Buenos Aires Province, Argentina.

**Other locality.** Mar del Plata (38°00'S, 57°33'W), Buenos Aires Province.

**Specimens in collections.** MACN No. 410/1 (holotype); MACN No. 410/2 (paratypes); USNPC No. 92399 (paratypes).

**GenBank Acc. Nos.**KP128023.

**References.**Ivanov and Brooks (2002), Alarcos et al. (2006), Bernot et al. (2015).

**Comments.** Specimens of *Calliobothriumeschrichti* van Beneden, 1850, identified by Ostrowski de Núñez (1973), were considered by Ivanov and Brooks (2002) as *C.barbarae*. Later, Bernot et al. (2015) transferred *C.barbarae* to the new genus *Symcallio*.

***Symcalliolunae* (Ivanov & Brooks, 2002)**

Calliobothriumlintoni Euzet, 1954, Calliobothriumlunae Ivanov & Brooks, 2002. Syns.

**Type host.***Mustelusschmitti* (Carcharhiniformes: Triakidae).

**Type locality.** La Paloma (34°40'S, 54°10'W), Rocha, Uruguay.

**Other locality.** Mar del Plata (38°00'S, 57°33'W), Buenos Aires Province.

**Specimens in collections.** MACN No. 411/1 (holotype); MACN Nos. 411/2–5 (paratypes); USNPC No. 92400 (paratypes).

**References.**Ivanov and Brooks (2002), Alarcos et al. (2006), Bernot et al. (2015).

##### ﻿Clade 4

#### Genus *Caulobothrium* Baer, 1948

***Caulobothriumostrowskiae* Brooks, Mayes & Thorson, 1981**

**Type host.***Myliobatisgoodei* (Myliobatiformes: Myliobatidae).

**Type locality.** Río de La Plata estuary, near Montevideo, Uruguay.

**Specimens in collections.**USNM No. 75726 (holotype); USNM No. 75727 (paratype), Univ. Nebraska State Museum No. 21004 (paratype).

**Reference.**Brooks et al. (1981).

***Caulobothriumuruguayense* Brooks, Mayes & Thorson, 1981**

**Type host.** Probably *Myliobatisgoodei* (Myliobatiformes: Myliobatidae) (*Myliobatisuruguayensis*).

**Type locality.** Río de la Plata estuary, Uruguay.

**Specimens in collections.**USNM No. 75724 (holotype); USNM No. 75725 (paratype); Univ. Nebraska State Museum No. 21002.

**Reference.**Brooks et al. (1981).

**Comments.***Caulobothriumuruguayense* was originally described by Brooks et al. (1981) from *Myliobatisuruguayensis*. However, this batoid’s name is invalid. Considering original article’s title, the type host of this cestode species is probably *M.goodei*.

##### ﻿Order Trypanoryncha Diesing, 1863

**Suborder Trypanobatoida Olson, Caira, Jensen, Overstreet, Palm & Beveridge, 2010**

**Superfamily Eutetrarhynchoidea Guiart, 1927**

#### Genus *Dollfusiella* Campbell & Beveridge, 1994

***Dollfusiellaacuta* Menoret & Ivanov, 2015**

**Type host.***Sympterygiaacuta* Garman (Rajiformes: Arhynchobatidae).

**Other hosts.***Atlantorajacastelnaui*, *Atlantorajaplatana*, *Sympterygiabonapartii* (Rajiformes: Arhynchobatidae).

**Type locality.** Off Punta Mejillón (41°11'S, 64°03'W), Río Negro Province, Argentina.

**Other localities.** off Puerto Quequén (38°37'S, 58°53'W), off Río Colorado (39°55'S, 62°03'W), Bahía Blanca, Buenos Aires Province. San Matías Gulf, Río Negro/Chubut Provinces.

**Specimens in collections.**MACN-Pa No. 575/1 (holotype); MACN-Pa Nos. 575/2–4 (paratypes); IPCAS No. C-700 (paratypes).

**References.**Menoret and Ivanov (2015), Irigoitia et al. (2017).

***Dollfusiellataminii* Menoret & Ivanov, 2014**

**Type host.***Psammobatisbergi* Marini (Rajiformes: Arhynchobatidae).

**Type locality.** Puerto Quequén (38°37'S, 58°53'W), Buenos Aires Province, Argentina.

**Other locality.** off Necochea (38°46'S, 57°56'W), Buenos Aires Province.

**Specimens in collections.**MACN-Pa No. 544/1 (holotype); MACN-Pa Nos. 544/2–4 (paratypes); IPCAS No. C-661 (paratypes).

**Reference.**Menoret and Ivanov (2014).

***Dollfusiellavooremi* (São Clemente & Gomes, 1989)**

*Eutetranychusvooremi* São Clemente & Gomes, 1989. Syn.

**Type host.***Musteluscanis* (Mitchill) (Carcharhiniformes: Triakidae).

**Other hosts.***Mustelusschmitti* (Carcharhiniformes: Triakidae).

**Type locality.** Southern Brazilian coast (30°40'S, 53°20'W–50°40'W).

**Other localities.** Off San Antonio Oeste (40°50'S, 64°58'W), Río Negro Province. Off Mar del Plata (38°00'S, 57°33'W), Buenos Aires Province.

**Specimens in collections.**CHIOC No. 32.566e (holotype); CHIOC Nos. 32.566a-d (paratypes); MACN-Pa Nos. 543/1–2 (vouchers).

**References.**São Clemente and Gomes (1989), Tanzola et al. (1998), Alarcos et al. (2006), Menoret and Ivanov (2014).

#### Genus *Mecistobothrium* Heinz & Dailey, 1974

***Mecistobothriumoblongum* Menoret & Ivanov, 2015**

**Type host.***Myliobatisgoodei* (Myliobatiformes: Myliobatidae).

**Type locality.** Off Punta Mejillón (41°11'S, 64°03'W), Río Negro Province, Argentina.

**Specimens in collections.**MACN-Pa No. 576/1 (holotype); MACN-Pa Nos. 576/2–3 (paratypes).

**Reference.**Menoret and Ivanov (2015).

#### Genus *Parachristianella* Dollfus, 1946

***Parachristianelladamiani* Menoret & Ivanov, 2014**

**Type host.***Myliobatisgoodei* (Myliobatiformes: Myliobatidae).

**Type locality.** Playa Punta Negra (38°36'S, 58°48'W), Necochea, Buenos Aires Province, Argentina.

**Specimens in collections.**MACN-Pa No. 545/1 (holotype); MACN-Pa No. 545/2 (paratypes), IPCAS No. C-660 (paratypes).

**Reference.**Menoret and Ivanov (2014).

##### ﻿Superfamily Tentacularoidea Poche, 1926

#### Genus *Heteronybelinia* Palm, 1999

***Heteronybeliniamattisi* Menoret & Ivanov, 2012**

**Type host.***Sympterygiabonapartii* (Rajiformes: Arhynchobatidae).

**Type locality.** Puerto Quequén (38°37'S, 58°53'W), Buenos Aires Province, Argentina.

**Specimens in collections.**MACN-Pa No. 537/1 (holotype); MACN-Pa Nos. 537/2–4 (paratypes); NHMUK Nos. 2012.9.11.1–2 (paratypes).

**Reference.**Menoret and Ivanov (2012a).

**Comments.** Larval stages (plerocercoids) of *H.mattisi* were reported from teleosts from coastal waters off Buenos Aires Province (Menoret and Ivanov 2012a).

##### ﻿Suborder Trypanoselachoida Olson, Caira, Jensen, Overstreet, Palm & Beveridge, 2010

**Superfamily Lacistorhynchoidea Guiart, 1927**

#### Genus *Grillotia* Guiart, 1927

***Grillotia*** (***Christianella***) ***carvajalregorum* Menoret & Ivanov, 2009**

*Progrillotiadollfusi* Carvajal & Rego, 1983, Grillotia (Progrillotia) dollfusi (Carvajal & Rego, 1983) Palm 2004, *Grillotiacarvajalregorum* Menoret & Ivanov, 2009. Syns.

**Type host.***Cynoscionstriatus* (Cuvier) (Perciformes: Sciaenidae).

**Other host.***Squatinaguggenheim* (Squatiniformes: Squatinidae).

**Type locality.** Coast of Brazil.

**Other locality.** Puerto Quequén (38°37'S, 58°53'W), Buenos Aires Province.

**Specimens in collections.**CHIOC No. 32.018a (holotype); CHIOC Nos. 32.018b–d (paratypes); MACN-Pa Nos. 487/1–2 (vouchers).

**References.**Carvajal and Rego (1983), Menoret and Ivanov (2009, 2012b), Beveridge and Campbell (2010).

**Comments.**Grillotia (C.) carvajalregorum was originally described from plerocercoids from *C.striatus* at coasts off Brazil (Carvajal and Rego 1983). Later, Menoret and Ivanov (2009) described adults of G. (C.) carvajalregorum from *S.guggenheim* at coasts of Argentina. This species was found in a wide range of teleost fishes (Menoret and Ivanov 2009, 2012b).

***Grillotia*** (***Grillotia***) ***patagonica* Menoret & Ivanov, 2012**

**Type host.***Psammobatisrudis* (Rajiformes: Arhynchobatidae).

**Other host.***Sympterygiabonapartii* (Rajiformes: Arhynchobatidae).

**Type locality.** Off Puerto San Julián (48°59'S, 65°15'W), Santa Cruz Province, Argentina.

**Other locality.** San Jorge Gulf, Santa Cruz Province.

**Specimens in collections.**MACN-Pa No. 534/1 (holotype); MACN-Pa Nos. 534/2–4 (paratypes).

**References.**Menoret and Ivanov (2012b), Irigoitia et al. (2017).

**Comments.**Grillotia (G.) patagonica was originally described from adults and plerocercoids caught at different localities along the Patagonian Shelf of Argentina (Menoret and Ivanov 2012b). Other reports in the area include this cestode in *S.bonapartii* at San Jorge Gulf (Irigoitia et al. 2017).

***Grillotia* sp.**

**Hosts.***Amblyrajadoellojuradoi*, *Bathyrajabrachyurops*, *B.cousseauae*, *B.griseocauda*, *Bathyrajamagellanica*, *Dipturuschilensis*, *Psammobatis* sp. 2, *Psammobatis* sp. 3.

**Locality.** Malvinas Islands Shelf, Southwestern Atlantic Ocean.

**Reference.**Beer et al. (2019).

**Comments.** These specimens were studied by Beer et al. (2019) at a molecular rather than a morphological level, without reaching a specific identification. Beer et al. (2019) noted the presence of *Grillotia* sp. parasitizing *D.chilensis*. However, the distribution of *D.chilesis* is restricted to the Pacific Ocean. Therefore, this record was based on a misidentified host (Concha et al. 2019).

### ﻿Species incertae sedis (at the ordinal level)

***Phyllobothriummyliobatidis* Brooks, Mayes & Thorson, 1981**

**Type host.***Myliobatisgoodei* (Myliobatiformes: Myliobatidae).

**Type locality.** Río de la Plata estuary, Uruguay.

**Specimens in collections.**USNM No. 1371266 (holotype); USNM No. 1371267 (paratype).

**Reference.**Brooks et al. (1981).

**Comments.**Ruhnke (2011) considers *P.myliobatidis* as a possible member of the order Rhinebothriidea.

## ﻿Host-parasite checklist: chimaeras and batoids

**Order Chimaeriformes**

**Family Callorhinchidae**

***Callorhinchuscallorynchus***

*Gyrocotylemaxima* (Gyrocotylidea)

*Gyrocotylerugosa* (Gyrocotylidea)

**Order Myliobatiformes**

**Family Myliobatidae**

***Myliobatisgoodei***

*Aberrapexarrhynchum* (Lecanicephalidea)

*Aberrapexludmilae* (Lecanicephalidea)

*Aberrapexsanmartini* (Lecanicephalidea)

*Acanthobothrium* sp. (Onchoproteocephalidea)

*Caulobothriumostrowskiae* (“Tetraphyllidea”)

*Caulobothriumuruguayense* (“Tetraphyllidea”)

*Halysioncummegacanthum* (Diphyllidea)

*Mecistobothriumoblongum* (Trypanorhyncha)

*Parachristianelladamiani* (Trypanorhyncha)

*Phyllobothriummyliobatidis* (Incertae sedis)

*Phyllobothrium* sp. (Phyllobothriidea)

***Myliobatisridens***

*Aberrapexvitalemuttiorum* (Lecanicephalidea)

**Order Rajiformes**

**Family Arhynchobatidae**

***Atlantorajacastelnaui***

*Acanthobothriummarplatensis* (Onchoproteocephalidea)

*Dollfusiellaacuta* (Trypanorhyncha)

*Notomegarhynchusnavonae* (Rhinebothriidea)

***Atlantorajaplatana***

*Dollfusiellaacuta* (Trypanorhyncha)

*Scalithriumivanovae* (Rhinebothriidea)

***Bathyrajaalbomaculata***

*Echeneibothrium* sp. (Rhinebothriidea)

*Rockacestusconchai* (Phyllobothriidea)

Phyllobothriidea gen. sp. (Phyllobothriidea)

***Bathyrajaarctowskii***

*Anthobothrium* sp. (“Tetraphyllidea”)

*Rockacestusarctowskii* (Phyllobothriidea)

***Bathyrajabrachyurops***

*Echeneibothrium* sp. (Rhinebothriidea)

*Grillotia* sp. (Trypanorhyncha)

*Guidusargentinense* (Phyllobothriidea)

Phyllobothriidea gen. sp. (Phyllobothriidea)

***Bathyrajacousseauae***

*Acanthobothrium* sp. (Onchoproteocephalidea)

*Echeneibothriumcristinae* (Rhinebothriidea)

*Echeneibothrium* sp. (Rhinebothriidea)

*Grillotia* sp. (Trypanorhyncha)

Phyllobothriidea gen. sp. (Phyllobothriidea)

***Bathyrajaeatonii***

*Guidusantarcticus* (Phyllobothriidea)

*Notomegarhynchusshetlandicum* (Rhinebothriidea)

*Onchobothriumantarcticum* (Onchoproteocephalidea)

*Pseudanthobothriumminutum* (Rhinebothriidea)

*Rockacestussiedleckii* (Phyllobothriidea)

***Bathyrajagriseocauda***

*Echeneibothrium* sp. (Rhinebothriidea)

*Grillotia* sp. (Trypanorhyncha)

***Bathyrajamaccaini***

*Guidusantarcticus* (Phyllobothriidea)

*Notomegarhynchusshetlandicum* (Rhinebothriidea)

*Rockacestusrakusai* (Phyllobothriidea)

***Bathyrajamacloviana***

*Echeneibothrium* sp. (Rhinebothriidea)

Phyllobothriidea gen. sp. (Phyllobothriidea)

***Bathyrajamagellanica***

*Acanthobothriumcarolinae* (Onchoproteocephalidea)

*Acanthobothrium* sp. (Onchoproteocephalidea)

*Grillotia* sp. (Trypanorhyncha)

*Guidusfrancoi* (Phyllobothriidea)

*Guidusmagellanicus* (Phyllobothriidea)

Phyllobothriidea gen. sp. (Phyllobothriidea)

***Bathyrajamultispinis***

*Echeneibothrium* sp. (Rhinebothriidea)

*Guidus* sp. (Phyllobothriidea)

Phyllobothriidea gen. sp. (Phyllobothriidea)

***Bathyrajascaphiops***

*Echeneibothrium* sp. (Rhinebothriidea)

Phyllobothriidea gen. sp. (Phyllobothriidea)

***Psammobatisbergi***

*Dollfusiellataminii* (Trypanorhyncha)

***Psammobatisnormani***

*Semiorbiseptumalfredoi* (Rhinebothriidea)

*Semiorbiseptummariae* (Rhinebothriidea)

***Psammobatisrudis***

Grillotia (G.) patagonica (Trypanorhyncha)

*Semiorbiseptummariae* (Rhinebothriidea)

***Psammobatis* sp. 1**

Rhinebothriidea gen. sp. (Rhinebothriidea)

***Psammobatis* sp. 2**

*Grillotia* sp. (Trypanorhyncha)

Phyllobothriidea gen. sp. (Phyllobothriidea)

Rhinebothriidea gen. sp. (Rhinebothriidea)

***Psammobatis* sp. 3**

*Grillotia* sp. (Trypanorhyncha)

Phyllobothriidea gen. sp. (Phyllobothriidea)

Rhinebothriidea gen. sp. (Rhinebothriidea)

***Riorajaagassizii***

*Scalithriumkirchneri* (Rhinebothriidea)

***Sympterygiaacuta***

*Dollfusiellaacuta* (Trypanorhyncha)

***Sympterygiabonapartii***

*Dollfusiellaacuta* (Trypanorhyncha)

Grillotia (G.) patagonica (Trypanorhyncha)

*Heteronybeliniamattisi* (Trypanorhyncha)

*Phyllobothrium* sp. (Phyllobothriidea)

*Rhinebothriumchilensis* (Rhinebothriidea)

**Family Rajidae**

***Amblyrajadoellojuradoi***

*Grillotia* sp. (Trypanorhyncha)

Phyllobothriidea gen. sp. (Phyllobothriidea)

*Pseudanthobothrium* sp. (Rhinebothriidea)

***Amblyrajageorgiana***

*Echinobothriumacanthocolle* (Diphyllidea)

*Pseudanthobothriumnotogeorgianum* (Rhinebothriidea)

*Rockacestusgeorgiensis* (Phyllobothriidea)

***Dipturusbrevicaudatus***

*Acanthobothriumdomingae* (Onchoproteocephalidea)

*Echeneibothriummultiloculatum* (Rhinebothriidea)

*Echeneibothriumwilliamsi* (Rhinebothriidea)

**Order Rhinopristiformes**

**Family Trygonorrhinidae**

***Zapteryxbrevirostris***

*Acanthobothriumzapterycum* (Onchoproteocephalidea)

*Acanthobothrium* sp. (Onchoproteocephalidea)

*Halysioncumpigmentatum* (Diphyllidea)

*Phyllobothrium* sp. (Phyllobothriidea)

**Order Torpediniformes**

**Family Narcinidae**

***Discopygetschudii***

*Acanthobothriumstefaniae* (Onchoproteocephalidea)

## ﻿Host-parasite checklist: sharks

**Order Carcharhiniformes**

**Family Carcharhinidae**

***Carcharhinusbrachyurus***

*Cathetocephalusaustralis* (Cathetocephalidea)

**Family Triakidae**

***Galeorhinusgaleus***

*Anthobothriumgaleorhini* (“Tetraphyllidea”)

***Mustelusfasciatus***

*Orygmatobothriumjuani* (Phyllobothriidea)

***Mustelusschmitti***

*Calliobothriumaustralis* (“Tetraphyllidea”)

*Coronocestusnotoguidoi* (Diphyllidea)

*Dollfusiellavooremi* (Trypanorhyncha)

*Orygmatobothriumschmitti* (Phyllobothriidea)

*Symcalliobarbarae* (“Tetraphyllidea”)

*Symcalliolunae* (“Tetraphyllidea”)

**Order Hexanchiformes**

**Family Hexanchidae**

***Notorynchuscepedianus***

*Crossobothriumantonioi* (Phyllobothriidea)

*Crossobothriumpequeae* (Phyllobothriidea)

**Order Squatiniformes**

**Family Squatinidae**

***Squatinaguggenheim***

Grillotia (C.) carvajalregorum (Trypanorhyncha)

*Paraberrapexatlanticus* (Lecanicephalidea)

### ﻿Geographical distribution of the cestode orders

**Figure 1.**
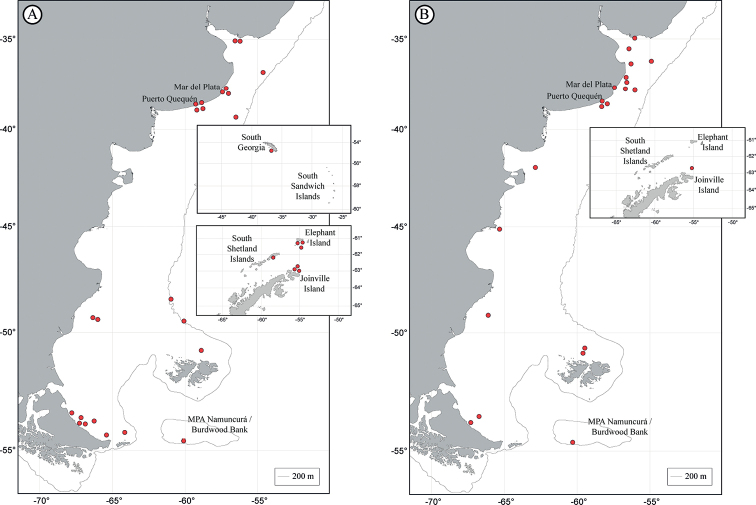
Distribution of representatives of the orders Phyllobothriidea and Onchoproteocephalidea**A** order Phyllobothriidea**B** order Onchoproteocephalidea. Insets show records in the sub-Antarctic and Antarctic regions.

**Figure 2.**
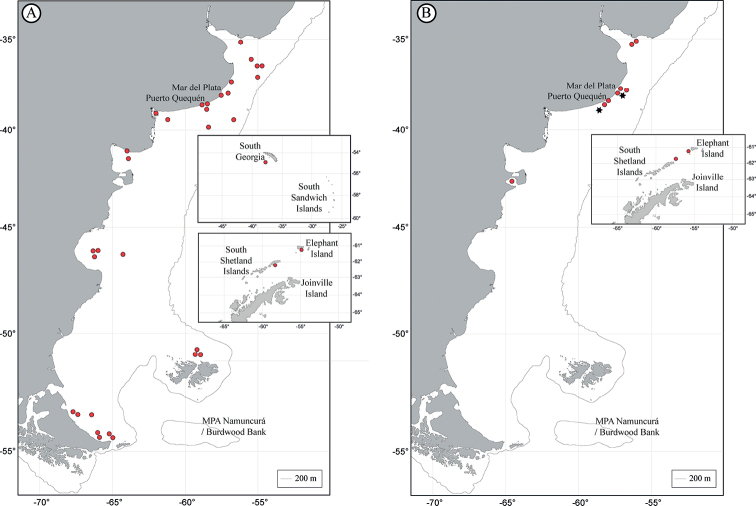
Distribution of representatives of the orders Rhinebothriidea, “Tetraphyllidea” and Gyrocotylidea**A** order Rhinebothriidea**B** red dot Orders “Tetraphyllidea” and black star Gyrocotylidea. Insets show records in the sub-Antarctic and Antarctic regions.

**Figure 3.**
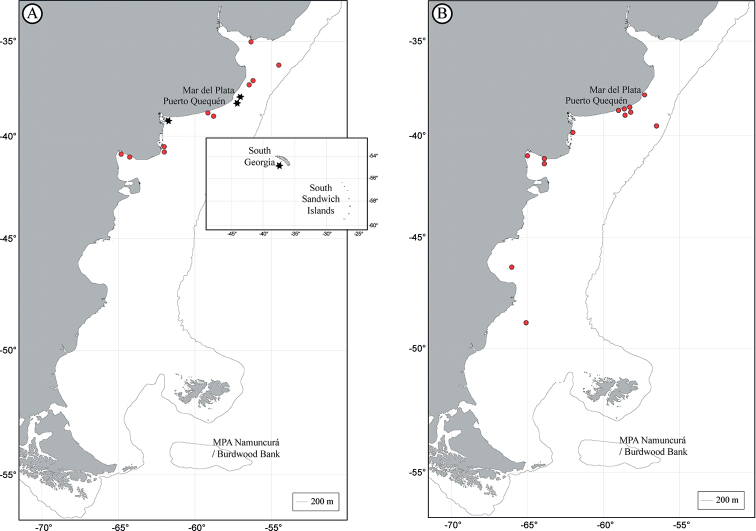
Distribution of the orders Diphyllidea, Lecanicephalidea and Trypanorhyncha**A** black star Diphyllidea and red dot Lecanicephalidea**B** order Trypanorhyncha. Inset shows records in the sub-Antarctic region.

The tapeworm orders reviewed in this study show different geographical ranges. These distributions are represented in Fig. 1A for the Phyllobothriidea, Fig. 1B for the Onchoproteocephalidea, Fig. 2A for the Rhinebothriidea, Fig. 2B for “Tetraphyllidea” and Gyrocotylidea, Fig. 3A for the Diphyllidea and Lecanicephalidea, and Fig. 3B for Trypanorhyncha. Geographical sites of the order Cathetocephalidea could not be represented since the only existing record reports *Cathetocephalusaustralis* in Argentina, without specifying the locality or coordinate.

The orders with the broadest geographic distributions are Onchoproteocephalidea (Fig. 1B) and Rhinebothriidea (Fig. 2A), with representatives in the Río de la Plata estuary, along the Argentine Sea, and the southern islands. In addition, the phyllobothriideans (Fig. 1A) show a similar distribution, although without records between the 40°S–47°S latitudes, in the central region of the Argentine Sea. On the other hand, the cestodes with the narrowest distribution are those of the order Gyrocotylidea (Fig. 2B), being recorded only in two locations in the Buenos Aires Province.

## ﻿Discussion

### ﻿Cestode diversity

Cestodes as parasites of chondrichthyans have been mostly recorded in the Northern Hemisphere (Caira et al. 2022). However, reports in southern latitudes have remarkably increased in the last decades due to focused sampling efforts in the area (Menoret and Ivanov 2012a, b, 2014, 2015, 2021; Pickering and Caira 2012; Caira et al. 2013a; Abbott and Caira 2014; Mutti and Ivanov 2016; Menoret et al. 2017; Franzese and Ivanov 2018, 2020a, b, 2021; Palm et al. 2019; Oosthuizen et al. 2021; Franzese et al. 2022; Van Der Spuy et al. 2022). The present annotated checklist comprises 57 valid cestode species of 28 genera in nine orders, registered in the Río de la Plata estuary, Southwestern Atlantic off Argentina and the surrounding waters off Antarctica. The orders Phyllobothriidea and Rhinebothriidea show the highest richness at the specific level, with 13 and 12 valid species, respectively; they are followed by the order Trypanorhyncha, with a total of eight species. In addition, the list includes cestodes without an identification up to the generic or the specific level, as in the case of the Onchoproteocephalidea (i.e., *Acanthobothrium* sp.), Phyllobothriidea (i.e., Genus sp., *Guidus* sp., *Phyllobothrium* sp.), Rhinebothriidea (i.e., Genus sp., *Echeneibothrium* sp., *Pseudanthobothrium* sp.) and Trypanorhyncha (i.e., *Grillotia* sp.) (Ostrowski de Núñez (1971); Brooks et al. 1981; Beer et al. 2019). Several of the comments made in this work are intended to aid in future morphological and molecular studies addressing the lower taxonomic resolution of these entities.

In view of the high degree of specificity of adult cestodes to their marine hosts (Reyda and Marques 2011; Caira and Jensen 2017) and that only 33% (33/100) of marine chondrichthyans in the study area have been sampled for cestodes (Table 1), this fauna is probably underestimated. We can speculate that more than 60 species of cestodes have not yet been discovered in this area. Future taxonomic surveys will be essential to increase the knowledge of the diversity of these parasites in the region.

### ﻿Taxonomic resolution

The poor taxonomic resolution of several taxa listed in the present study is probably a consequence of the lack of use of multiple tools to develop an integrative taxonomy, such as morphological and molecular studies used as evidence to delineate species boundaries. Some of the previous works cited here lacked modern morphological tools, e.g., scanning (SEM) and transmission electron microscopy (TEM) and the molecular tools necessary for the development of an integrative approach (Ostrowski de Núñez (1971); Brooks et al. 1981). In contrast, the recent work by Beer et al. (2019) recorded in the Argentine Sea numerous specimens of cestodes belonging to different orders but without achieving a specific identification for many of them, using molecular sequences as the only identification tool. The development and use of molecular tools have allowed the detection of cryptic species in some cestode groups (Scholz et al. 2014; Choudhury and Scholz 2020). Of the 57 valid species recorded in this work, only seven have been sequenced, so it is still unknown whether cryptic species will be discovered in this particular region. In addition to molecular sequences, the use of modern morphological tools, such as SEM and TEM, might be helpful in discovering new characters that complement traditional morphological studies, which could contribute to solve species identification problems (Franzese et al. 2023; Mutti et al. 2023). The development of the integrative taxonomy, including the use of all available tools, will allow resolving the poor taxonomic resolution observed in several taxa registered in our study area.

### ﻿Host association

Rajiform batoids represent the most frequent hosts for adult cestodes in the study area. In particular, the family Arhynchobatidae has been found parasitized by 42% (24/57) of the recorded cestode species (Ivanov and Campbell 1998b, 2002; Rocka 2003; Menoret and Ivanov 2012a, 2014, 2021; Irigoitia et al. 2017; Franzese and Ivanov 2020a, b; Caira et al. 2021; Franzese et al. 2022). This percentage could be higher since many species of arhynchobatids of the region, such as *Atlantorajacyclophora* (Regan), *Bathyrajameridionalis* Stehmann, *Bathyrajapapilionifera* Stehmann, *Bathyrajaschroederi* (Krefft), *Psammobatisextenta* (Garman), *Psammobatislentiginosa* McEachran, *Psammobatisparvacauda* McEachran, *Psammobatisrutrum* Jordan, and *Psammobatisscobina* (Philippi), have not yet been sampled for cestodes. The association between tapeworms and this host family is not surprising if we consider that arhynchobatids have the highest species number, with 31% (31/100) of the chondrichthyan fauna recorded in the area (Table 1) (Menni and Lucifora 2007; Froese and Pauly 2022). Upcoming studies should focus on sampling arhyncobatids that have not yet been reported as hosts for tapeworms.

Considering that the major number of cestode species from this checklist are hosted by the myliobatiform *Myliobatisgoodei* (Brooks et al. 1981; Ivanov and Campbell 1998a; Menoret and Ivanov 2014, 2015; Menoret et al. 2017), it would be interesting to sample *M.freminvillei* Lesueur, the only species of myliobatid that has not been yet examined for cestodes in the region. On the other hand, only 13% (6/45) of the species of sharks have been reported as hosts in this area (Table 1). Host species with a relatively low occurrence or a particular bathymetric distribution are likely to host an undiscovered and exciting cestode fauna.

More collecting efforts are necessary to conclude if this data reflects the actual biodiversity of cestodes in the different groups of chondrichthyans or is a result of a bias in sampling. Although this list shows the substantial advances in taxonomical surveys in the last decades, expanding the number of sampled hosts is essential to increase the knowledge of the current cestode fauna of chondrichthyans in the region.

### ﻿Studied area and newly collected material

Five species of cestodes have been recorded in new localities of the Southwestern Atlantic Ocean (Table 3). New material (voucher) identified, processed, and deposited in the MACN parasitological collection corresponds to three onchoproteocephalideans (i.e., *Acanthobothriumdomingae*, *A.marplatensis*, *A.stefaniae*) and two rhinebotriideans (i.e., *Echeneibothriumwilliamsi*, *Notomegarhynchusnavonae*). One of these records has extended until the Buenos Aires Province the northern limit of the known geographic distribution in the Argentine Sea of *E.Williamsi*, which, prior to this work, ranged from Santa Cruz Province to Tierra del Fuego Province (Franzese et al. 2022). The remaining new records have added new localities within the province of Buenos Aires for *A.domingae*, *A.marplatensis*, *A.stefaniae*, and *N.navonae*. Previously, these four species had been reported off Buenos Aires, although in different locations (Ivanov and Campbell 1998b, 2002; Franzese and Ivanov 2018, 2020a).

Several of the original descriptions of cestode species are based on material collected from a single locality. However, this probably reflects the absence of a more exhaustive sampling. The present checklist shows that about half of the species included in this region have additional localities. Among these, *Rhinebothriumchilensis* and *Echeneibothriumwilliamsi* show the highest number with 7 and 6 localities, respectively (Tanzola et al. 1998; Irigoitia et al. 2017). It is likely that as the intensity of sampling increases, new localities will be discovered for several known cestode species.

The localities with the most significant number of cestodes species are Puerto Quequén and Mar del Plata, with 17 and 11 species reported to date, respectively. A strong sampling effort could explain these numbers since both sites are commercial ports from the Buenos Aires Province close to the facilities of the main Argentinean research taxonomic cestodes groups (Luque and Poulin 2007; Randhawa and Poulin 2019).

## ﻿Conclusions

Some difficulties concerning the understanding of chondrichthyan cestode diversity are:

1. many works have a poor taxonomic resolution or are outdated, with incomplete drawings and without the use of modern tools such as transmission electron microscopy, scanning electron microscopy and molecular approaches;
2. the existence of cryptic species underestimates the actual number of cestodes;
3. less than half of the marine chondrichthyans have been examined for cestodes in the area covered in this work.

A modern taxonomic approach for future characterizations should be made by combining descriptive tools (e.g., TEM and SEM, molecular data, histological sections, and histochemical techniques). It would also be desirable that all the voucher material could be available in public parasitological collections to facilitate its study to the entire community of taxonomists. Regarding sampling effort, it is likely that the higher the number of chondrichthyans examined in parasitological surveys, the higher the number of parasite-host associations will be identified. We have critically compiled as much detailed information as possible including valuable comments, providing a complete list of references and information from the deposited material. We hope this list may help future studies and contributes to correctly estimating the cestode biodiversity that inhabits this underexplored region of the Southern Hemisphere.
